# The immunology of sickness metabolism

**DOI:** 10.1038/s41423-024-01192-4

**Published:** 2024-08-06

**Authors:** Felix M. Wensveen, Marko Šestan, Bojan Polić

**Affiliations:** University of Rijeka Faculty of Medicine, Rijeka, Croatia

**Keywords:** Immunometabolism, infection, metabolic disease, immune system, inflammasome, Infection, Infectious diseases

## Abstract

Everyone knows that an infection can make you feel sick. Although we perceive infection-induced changes in metabolism as a pathology, they are a part of a carefully regulated process that depends on tissue-specific interactions between the immune system and organs involved in the regulation of systemic homeostasis. Immune-mediated changes in homeostatic parameters lead to altered production and uptake of nutrients in circulation, which modifies the metabolic rate of key organs. This is what we experience as being sick. The purpose of sickness metabolism is to generate a metabolic environment in which the body is optimally able to fight infection while denying vital nutrients for the replication of pathogens. Sickness metabolism depends on tissue-specific immune cells, which mediate responses tailored to the nature and magnitude of the threat. As an infection increases in severity, so do the number and type of immune cells involved and the level to which organs are affected, which dictates the degree to which we feel sick. Interestingly, many alterations associated with metabolic disease appear to overlap with immune-mediated changes observed following infection. Targeting processes involving tissue-specific interactions between activated immune cells and metabolic organs therefore holds great potential for treating both people with severe infection and those with metabolic disease. In this review, we will discuss how the immune system communicates in situ with organs involved in the regulation of homeostasis and how this communication is impacted by infection.

## Introduction

**Fig. 1:**
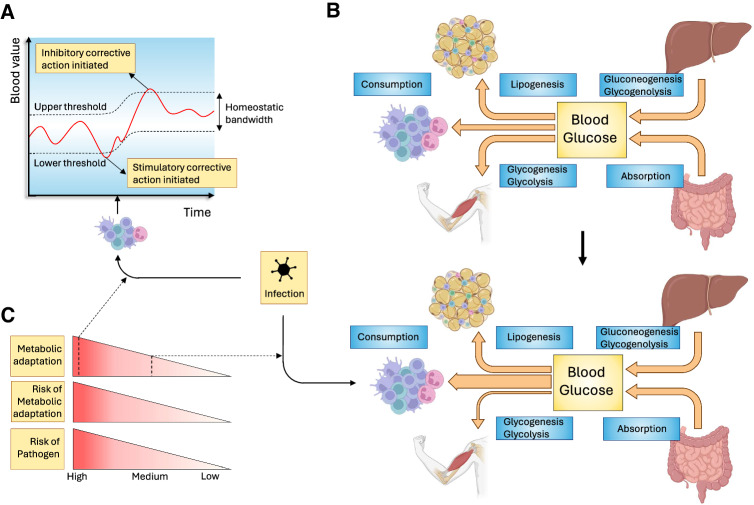
Impact of infection on nutrient homeostasis. Infection can impact the regulation of systemic homeostasis in several ways. **A** Values of various parameters in the blood are maintained between threshold values. If blood values are outside of this homeostatic bandwidth, corrective action is undertaken. One way by which the immune system can mediate sickness metabolism following infection is by altering these threshold values. **B** Blood parameter homeostasis is regulated through a dynamic balance between the influx and efflux of nutrients. In this figure, blood glucose is given as an example. Infection can alter this balance in several ways. If threshold values are not altered, changes in nutrient flux are possible only if they are compensated for by equal and opposite alterations in other organs involved in maintaining homeostasis. In this example, reduced glucose uptake by skeletal muscle is compensated for by an increased flux of glucose to the immune system. **C** Changes in homeostatic regulation negatively impact normal organ function and are therefore not without risk. Strategies with a greater impact, such as changes in threshold levels, are therefore justified only if the risk of serious damage by the pathogen is high. Threats of a lower danger level will therefore have less impact on homeostasis, for example, by altering nutrient flux toward a single organ. Made with biorender.com

In the absence of infection, blood levels of key metabolic factors such as nutrients, electrolytes and vitamins are kept within a strictly regulated range of threshold values, a process also known as homeostasis [1]. Homeostasis ensures that all organs can perform highly specialized functions without the need to invest resources in generating the specific building blocks required for those tasks [1]. Homeostasis is regulated at multiple levels, but its systemic coordination is ensured through the continuous measurement and corrective action of the nervous and endocrine systems working in tandem [2, 3]. By providing activating or inhibitory signals, these systems ensure that homeostasis is maintained despite continuous changes in the influx and efflux of nutrients, for example, following feeding and fasting. Importantly, by controlling the flux of nutrients, the metabolic rate of cells and organs can be controlled [1]. One process that has a major impact on the normal control of homeostasis is infection. Following the encounter of a pathogen, the activated immune system mediates many changes to local and systemic metabolism, a process that we experience as being sick [4]. These changes are mediated at two levels: 1. an altered contribution of organs to the regulation of homeostatic set points [5] or 2. an alteration of homeostatic set points. The former is used to redirect nutrients from one organ system to another, whereas the latter alters the systemic availability of metabolites (Fig. 1A, B).

Infection-induced changes in metabolism are typically perceived as a pathology since they make people feel sleepy, weak and fatigued. However, these changes are highly conserved mechanisms that benefit the antipathogenic response. For example, both mice and humans show changes in the way blood glucose is regulated following viral infection, which alters their systemic sensitivity to insulin [6, 7]. Mice that fail to induce insulin resistance have a reduced CD8 + T-cell response, resulting in higher viral titers. Fever is a well-known symptom of both viral and bacterial infection, and its prevention by antipyretic drugs increases mortality in patients infected with influenza virus [8, 9]. The evolutionary benefit of an increased body temperature during infection is illustrated by the fact that this state is induced even in cold-blooded (ectothermic) animals. Ectothermic animals, including fish, reptiles and insects, some of which have their last common ancestor with mammals more than half a billion years ago, seek warmer environments to increase their core temperature [10–12]. It was shown that if iguana are prevented from raising their body temperature, lethality greatly increases following bacterial infection [12]. Another example of conserved sickness behavior is anorexia, or a loss of appetite. Anorexia forces the body to use energy reserves stored in fat rather than from nutrition. However, a reduction in nutrient intake is observed in organisms as diverse as humans, mice and insects such as *Drosophila melanogaster* [13, 14]. Notably, gavage feeding of mice increased lethality in those given a sublethal dose of *Listeria monocytogenes* [15, 16]. Fruit flies show a greater mortality rate following *M. luteus* infection when provided a diet rich in proteins [13]. Metabolic changes in response to infection put a heavy strain on the energy reserves of the body. For example, fever requires a 10–12.5% increase in metabolic rate for every degree Celsius that the core temperature is increased [17]. Nevertheless, the evolutionary conservation of these changes indicates that they have a major survival benefit. We therefore want to coin the term ‘sickness metabolism’ to emphasize that infection-induced metabolic changes are deliberate modifications that aim to benefit the antipathogenic response.

It is important to realize that infection-induced changes in metabolism come at a price to the normal biology of the organism. If glucose is redirected away from muscle, this means that this organ can function only suboptimally. As a result, infection can lead to lethargy and asthenia [18]. This tradeoff is acceptable during an infection, since feeding the immune system has a higher priority than motility when warding off a potentially lethal pathogen. Indeed, physical activity alters the immune system [19], and forced exercise during infection increases morbidity and mortality in animals infected with influenza virus [20, 21]. The price of sickness metabolism is even greater when systemic metabolic threshold values are altered, as this will affect all systems in the body that are dependent on that factor. In the case of fever, a higher temperature promotes immune cell function [22, 23]. However, a higher temperature also dramatically reduces the half-life of proteins; therefore, increased proteosynthesis is required. Since this is a highly energy-intensive process, fever leads to a major increase in energy expenditure [24]. Changing set points is therefore a coping strategy with a higher cost and risk profile and therefore is typically implemented only when the threat to survival is sufficiently high, i.e., when the infection is of greater severity or reaches vital organs (Fig. 1C).

Systemic homeostasis is predominantly regulated by the endocrine and nervous systems. However, in the case of infection, there must be an additional system that evaluates the threat and determines whether sickness metabolism should be initiated at the organ or systemic level. Virtually all cells in the body have the capacity to sense tissue damage and pathogenic components and to communicate this information to other cells, for example, through the production of type I interferons [25]. However, only the immune system can properly evaluate the nature and magnitude of the threat and the requirement of a small-scale local or large-scale systemic response. Every organ in the body therefore has its own tissue-resident population of immune cells to fine-tune this response. Under normal conditions, this immune cell pool is dominated by anti-inflammatory cells, such as regulatory T cells, M2-like macrophages and type 2 innate lymphoid cells (ILC2s). These cells maintain homeostasis through the secretion of anti-inflammatory cytokines such as IL-10 [26]. When an organ is infected, pathogen- and danger-associated molecular patterns (PAMPS and DAMPS, respectively) are detected by tissue-resident immune cells. These cells are then activated and attempt to resolve the threat of disrupted tissue homeostasis. If they cannot, these immune cells increase their response and produce signals to recruit bone marrow-derived innate immune cells, initially neutrophils and proinflammatory macrophages. After several days, signals from the infected site lead to the recruitment of lymphocytes activated in draining lymph nodes. If this still does not resolve the infection, the response is escalated to a systemic level and an organism-wide response is warranted [4].

Apart from their obvious role as direct responders to infection in situ, the added benefit of tissue-resident immune cell pools is that they allow the fine-tuning of metabolic adaptations within and between organs according to the necessary scale of escalation. For example, local infection of the skin causes well-known symptoms associated with inflammation, such as redness, pain, swelling and heat. When infection is minor, it causes only small metabolic changes in cells at the site of pathogen entry, for example, by increasing the uptake of glucose and amino acids by keratinocytes [27]. If the infection spreads further through an organ, larger metabolic adaptations are induced that are still restricted in scope. The reduced insulin sensitivity of skeletal muscle cells following infection, without changes in systemic glucose levels, is an example of such an escalated yet still limited metabolic change [6]. If this strategy is still insufficient and the threat spreads beyond the initially infected organ, increasingly debilitating strategies of sickness metabolism are implemented, including anorexia, lethargy, fever and immobilization [4]. When pathogenic loads reach critical levels, such as during sepsis or severe SARS-CoV-2 infection, stress hormones such as cortisol are produced by the adrenal glands. These stress hormones lead to metabolic adaptations such as stress-induced hyperglycemia, aiming to maintain the function of vital organs under extreme conditions [28, 29].

In summary, metabolic changes are an integral part of the response against infection. The type and intensity of sickness metabolism are tailored to the level of the threat and depend on signals from tissue-resident immune cells. Below, we will elucidate how infection impacts key organ systems involved in the regulation of metabolic homeostasis.

## Skeletal muscle

By mass, skeletal muscle comprises a large fraction of the human body. Skeletal muscle therefore has a major impact on the homeostasis of nutrients: 1. as a drain of nutrients needed for protein synthesis and movement [30, 31], 2. as a sink for metabolites such as glucose upon eating a meal [32] and 3. as a source of lactate following anaerobic exercise and glucose absorption [33, 34]. Not surprisingly, skeletal muscle is an important target of immune-mediated metabolic modulation under several different conditions. During homeostasis, skeletal muscle is populated with a plethora of immune cells [35, 36], most notably of the myeloid lineage [37]. Among mononuclear immune cells, macrophages make up the majority. The transcriptional profile of macrophages indicates that many of these cells have functions associated with the removal of cellular debris and tissue repair. In addition, some populations of muscle macrophages have a marked proinflammatory phenotype [36, 37]. Muscle damage following trauma or intense exercise has been shown to lead to the secretion of cytokines, most notably IL-6 [38]. The ensuing inflammation is required for proper activation of muscle satellite cells and the regeneration of muscle fibers [39]. Tissue damage therefore leads to a dramatic increase in immune cells in skeletal muscle [37], and the abrogation of the inflammatory response significantly hampers the rate of tissue repair [40].

In the context of infection, the body reprioritizes its usage of nutrients. Various well-known physiological mechanisms are triggered that minimize the metabolic strain of skeletal muscle on the body, for example, by promoting lethargy and sleep [41]. In addition, resident immune cells directly modulate the metabolic activity of muscle. Mild infection causes insulin resistance in skeletal muscle cells in both humans and mice [7, 26, 42]. Upon viral infection, NK cells in skeletal muscle secrete IFNγ, which directly targets skeletal muscle cells [6]. As a result, myocytes downregulate their insulin receptor expression, making them less sensitive to this mediator of systemic glucose levels. In the absence of preexisting metabolic aberrations, infection does not result in hyperglycemia because the pancreas compensates for skeletal insulin resistance by secreting more insulin. Thus, mild infection causes a state of euglycemic hyperinsulinemia [42], which promotes antiviral T-cell responses. The receptor for insulin is expressed on many cells, including T cells. Ligand engagement by the insulin receptor leads to the activation of PI3K, which is a molecule that is also activated by key activating receptors on T cells, such as the costimulatory molecule CD28. Insulin therefore directly promotes T-cell function, and insulin deficiency impairs antiviral CD8 + T-cell responses, whereas its exogenous administration to mice stimulates antigen-specific T-cell responses [6, 43]. In addition, increased insulin resistance in skeletal muscle redirects nutrients to other organs and systems, most notably to the immune system, which is a major drain of resources during infection [44]. Infection-induced modulation of skeletal muscle metabolism modifies the composition of nutrients in the bloodstream, most notably of lactate. Following exercise but also in response to a glucose-rich meal, skeletal muscle rapidly converts glucose into lactate, which is deposited in the bloodstream [33]. Lactate is a natural inhibitor of MAVS-mediated sensing of viral genomes and drives the production of IFN-I [45]. Viruses are therefore known to promote the expression of lactate dehydrogenase in infected cells to stimulate lactate production and inhibit MAVS-dependent IFN-I production [46, 47]. By redirecting glucose from skeletal muscle toward other organs [48], the immune system reduces its contribution to systemic lactate levels and thus its inhibitory impact on IFN-I production. Therefore, the inhibition of this mechanism impairs antiviral control [45–48].

The result of infection-induced metabolic changes to skeletal muscle is reduced strength. A meta-analysis including 168 studies and 89,194 patients found that muscle strength was negatively correlated with serum levels of several proinflammatory cytokines, most notably CRP, IL-6 and TNF [49]. Subgroup analysis revealed that muscle strength loss was strong in patients with viral infections, including cytomegalovirus (CMV) and HIV. This group did not show changes in muscle mass, suggesting altered metabolic activity of this tissue rather than a loss of fibers [49]. Mouse studies confirmed that both viral and bacterial infection promote the influx of immune cells in muscle tissue, resulting in a reduction in grip strength [50–52]. Thus, during infection, sickness metabolism redirects nutrients from skeletal muscle at the price of reduced functionality of this tissue.

The metabolic adaptation of skeletal muscle tissue to infection can become detrimental under chronic or severe conditions. Sarcopenia is a loss of muscle strength, usually without a loss of tissue mass. This condition is frequently observed in elderly individuals and is subsequently associated with the senescence of muscle fibers [53]. Sarcopenia has a strong immunological component, and cytokines such as IL-6, TNF and IL-1β are known to aggravate this condition in elderly individuals [53, 54]. These cytokines are also abundantly produced following viral infection, and indeed, sarcopenia has been frequently observed in patients infected with viruses such as SARS-CoV-2 and HIV but also following sepsis [55–57]. mTOR signaling is crucial for maintaining skeletal muscle mass [58]. For example, during sepsis or after cytomegalovirus infection [59, 60], mTOR signaling was shown to be deregulated in muscle cells, causing myofiber denervation, oxidative stress and a deregulation of protein degradation, resulting in a loss of muscle function [61–63]. Cachexia, or wasting disease, is the loss of adipose and muscle tissue mass because of systemic metabolic changes [64]. A condition typically associated with the late stages of cancer or severe infection, cachexia causes the body to use lipid stores instead of glucose as a primary source of catabolic fuel [65]. Once adipose nutrient stores are depleted, muscle protein is degraded as the carbon molecules from amino acids are converted into energy [64]. This process is the result of metabolic reprogramming by the immune system and therefore cannot be prevented by parenteral feeding [66]. Animals infected with a high dose of lymphocytic choriomeningitis virus showed considerable signs of cachexia, which was prevented in animals deficient in the type I interferon receptor. Type I interferons promote cachexia via T cells, as animals with T cell-specific deficiency of the type I interferon receptor failed to develop infection-induced cachexia [67].

In summary, immune cells inside skeletal muscle directly modify the metabolism of myocytes during infection, the purpose of which is to regulate systemic levels of metabolites such as glucose, amino acids, insulin and lactate, which help the immune system better fight infection.

## Adipose tissue

Adipocytes are the main carbohydrate storage vessels in the human body. Adipose tissue can be subdivided into white adipose tissue (WAT), which predominantly stores lipids in a single large lipid droplet in adipocytes, and brown adipose tissue (BAT), in which adipocytes contain multiple lipid droplets and generate heat [68]. The metabolism of both brown and white fat is subject to changes in response to infection. For example, BAT plays an important role in mediating fever, especially in rodents [69]. However, BAT appears to be less subject to immunological control in situ, which is why we will focus on WAT here. WAT is under tight immunological control for three reasons. First, because WAT contains many nutrients, it must be shielded from pathogens aiming to exploit its anabolic potential [70–72]. Second, the regulation of the influx and efflux of nutrients from WAT allows the control of systemic metabolism. If more lipids are available in circulation, tissues will depend less on glucose and glutamine to fulfill their energetic needs [33]. The modulation of WAT biology therefore allows the adjustment of systemic metabolite threshold values and is thus a key modulator of sickness metabolism [42]. In addition, WAT regulates systemic metabolism through the secretion of hormones called adipokines. These hormones communicate the status of nutrient availability, promoting metabolism when adipocytes are filled with fat and inhibiting it when they are depleted [73]. Third, infection is typically associated with major changes in nutrient consumption, for example, by immunological organs [6, 14, 74]. Increased nutrient efflux from WAT, which is partially regulated by the immune system, is required to accommodate these altered metabolic needs.

In the absence of infection, the immune cell pool in WAT is dominated by immune cells with an anti-inflammatory profile, including adipose tissue macrophages (ATMs) with predominantly an M2-like phenotype, ILC2s, regulatory T cells and NKT cells [26]. These cells ensure tissue homeostasis through the secretion of cytokines such as IL-4, IL-5, IL-13 and IL-10. If the function of these cells is abrogated, for example, through a genetic inability of ATMs to respond to M2-polarizing signals, inflammation is induced, leading to an increase in proinflammatory cytokines such as TNF and IL-1β in adipose tissue [75]. Much is known about the role of WAT-resident inflammatory cells in the context of obesity [26]. Surprisingly, much less is clear about how the immune system interacts with WAT in the context of infection [7]. However, in general, infection appears to induce a net efflux of nutrients from adipose tissue [76–78]. Infection with various pathogens causes an acute, transient increase in serum triglyceride levels [76–78]. More recently, serum triglycerides were shown to be positively correlated with the severity of SARS-CoV-2 infection [79]. Infection-induced nutrient release from adipocytes appears to be mediated by tissue-resident immune cells. Following helminth infection of the small intestine, stromal cells in adjacent adipose tissue start producing interleukin-33 (IL-33) and thymic stromal lymphopoietin (TSLP). These signals recruit T helper 2 cells, which mediate stromal reprogramming and a decrease in adipose tissue mass [80]. The infection of mice with *T. gondii* or *Y. pseudotuberculosis* was found to cause the accumulation of large numbers of pathogen-specific memory T cells with a unique transcriptional profile in WAT [70]. These cells were functionally potent, produced high levels of IFNγ and TNF and efficiently protected animals from secondary infection. In addition, the stimulation of adipose tissue-resident T cells mediated metabolic changes in WAT, shifting the balance from lipogenesis to lipolysis [70]. As a result, the nutrient content of WAT decreased. During severe infection, this system appears to derail, leading to rapid depletion of adipose tissue reservoirs and the induction of cachexia, which is mediated by CD8 + T cells [67].

Following infection, immune cells can also mediate changes in the characteristics of WAT. White adipose tissue has the capacity to gain properties of brown fat, such as the expression of uncoupling protein 1 (UCP1), a molecule that mediates heat generation in brown adipose tissue. This process, also referred to as ‘browning’, was shown to be mediated by the cytokine IL-33 produced by type 2 innate lymphoid cells [81] and occurred following infection with viral pathogens such as SARS-CoV-2 and influenza A virus [82, 83]. Since immune cells are highly sensitive to thermal stress [84, 85], this metabolic adaptation to infection was proposed to increase body temperature to promote the antiviral response [82]. Interestingly, some viruses appear to cause increased adiposity [86]. Canine distemper virus was shown to cause obesity in mice [87]. This effect was not mediated through the interaction of immune cells with adipose tissue but rather through the infection of neurons and glial cells in the brain. Damage to the hypothalamus caused by this virus was shown to promote nutrient uptake in adipose tissue and thus cause obesity [87]. Similar observations were made for scrapie, a neurodegenerative prion disease, and Rous-associated virus-7 in chickens [88, 89]. Additionally, these pathogens cause nutrient accumulation in adipocytes indirectly through the upregulation of GLUT-1 transporters on sensory neurons in the hypothalamus [89] and through the modification of thyroid hormone levels [88]. Potentially, virus-mediated adipogenesis is an immune-evasive strategy aimed at generating a suboptimal systemic environment for antipathogenic responses. In humans, no direct evidence has shown that infection can cause obesity. Obese individuals were shown to have more antibodies against pathogens such as adenovirus-36 [90]. However, since obesity negatively impacts the antiviral immune response [7, 91], it is questionable whether a history of more frequent viral infection is the cause or effect of this condition. In humans, the amount of adipose tissue around the intestines appears to increase following the penetration of bacteria into the intestinal wall [92], a process known as ‘creeping fat’. However, the purpose of creeping fat appears to be to form an extra protective layer around the intestines to protect against the spread of pathogens rather than adipose reprogramming to alter systemic metabolism [92].

In summary, infection causes a change in adipose tissue biology, which is mediated by the immune system. These changes are proposed to aid the systemic response against pathogens by increasing lipid availability and modifying body temperature.

## Endocrine glands—The pancreas and adrenal glands

The pancreas plays a key role in transforming the food that we eat into the metabolites that we need to sustain our body. The exocrine pancreas produces various factors, most notably enzymes, that contribute to the digestion of food. The endocrine pancreas produces hormones such as insulin and glucagon, which control the homeostasis of metabolites such as glucose in the blood, as well as the activity of the exocrine pancreas [93, 94]. Clearly, the pancreas is of great importance for maintaining metabolic homeostasis, and not surprisingly, the pancreas, especially the Langerhans islets, is extensively surveilled by the immune system. Langerhans islets, as regulatory nexuses, contain a plethora of immune cells that provide an important defense against infection and aid in tissue healing after injury [95]. The most abundant immune cells inside islets are anti-inflammatory M2 macrophages [96]. These cells can be divided into CD11c^+^ F4/80^+^ intra-islet and CD11c^+^ F4/80^high^ peri-islet cells [97]. Langerhans islets also contain a relatively large pool of B cells, T cells, dendritic cells (DCs) and type 2 innate lymphoid cells (ILC2s), whereas NK cells and granulocytes are quite rare [96, 98].

Apart from protecting the pancreas against infection, immune cells within islets also modulate systemic metabolism by impacting their endocrine function even in the absence of infection. This phenomenon has been investigated most thoroughly for insulin production. Upon feeding, macrophages are triggered to produce IL-1β, which directly stimulates β cells and promotes their production of insulin following glucose exposure. Moreover, genetic ablation of IL-1β decreases the levels of insulin in serum [99]. Additionally, macrophages are essential for normal β-cell development, and macrophage depletion impairs insulin production [97, 100]. A second subset of islet-resident immune cells that impact insulin production are type 2 innate lymphocytes (ILC2s). Mesenchymal cells in Langerhans islets produce IL-33, which promotes the production of IL-13 and CSF2 by ILC2s. In turn, these cytokines stimulate macrophages and DCs to produce retinoic acid (RA), which is essential for glucose-stimulated insulin secretion [98]. The injection of recombinant IL-33, a potent activator of ILC2s, in mice stimulates insulin production and lowers blood glucose levels [98]. Surprisingly, the impact of immune signals on the function of other pancreatic endocrine cells is almost completely unexplored. To date, only the proinflammatory cytokine IL-6 has been shown to stimulate glucagon production [101].

The extent of infection-induced changes in hormone production by the pancreas depends on the intensity of infection and the levels of cytokines in circulation. Following mild infection, β-cell responsiveness to glucose is not altered. However, because infection causes insulin resistance in skeletal muscle cells, the pancreatic insulin output is increased to compensate for the reduced glucose uptake by the muscle [102]. Only when cytokine levels reach a certain concentration does the sensitivity of pancreatic β cells increase and glucose threshold values decrease. β cells express the IL-1β receptor, and in vitro stimulation of pancreatic islets promotes their production of insulin [103]. In vivo, the injection of animals with LPS, as a model for sepsis, was shown to promote insulin production in an IL-1β-dependent fashion [104]. Notably, infection with a high viral dose lowered blood glucose levels [105–107], which was dependent on cytokines such as IL-1β, IL-1α, IFNγ and TNF and proposed to be the result of hyperinsulinemia [108]. Infection-induced hypoglycemia seems to have a beneficial role in the antiviral host response through the restriction of glucose availability to the pathogen [42, 93]. Indeed, the replication of viruses such as SARS-CoV-2 and influenza is impaired in cells treated with 2-deoxyglucose, which blocks the entry of glucose into glycolysis [109, 110]. Conversely, hyperglycemia induced by the elimination of pancreatic β cells increases viral titers and mortality following infection of mice with Dengue virus, influenza virus and coxsackievirus [111–113]. Infection-induced glucose restriction requires immunological signals both in situ and from the periphery. In a recent manuscript, it was reported that IL-1β produced by macrophages inside Langerhans islets in response to strong infection was not sufficient to induce hyperinsulinemia; a systemic surge of IFNγ was also required [47]. The insulin output of purified pancreatic β cells increased only when they were simultaneously stimulated with both IL-1β and IFNγ [47]. Interestingly, the stimulation of pancreatic islets with very high concentrations of TNF, IFNγ and IL-1β inhibited insulin secretion, indicating that during severe, life-threatening infection these cytokines are involved in facilitating stress-induced hyperglycemia [114–116].

The adrenal gland is another important regulator of systemic homeostasis. The cortex of the adrenal gland produces steroid hormones that regulate electrolyte balance, gonad activity and metabolism, whereas the medulla produces adrenalin and noradrenalin. The cortex of the adrenal gland is part of the hypothalamo-pituitary-adrenal (HPA) axis. HPA axis activity is induced by hormones [112] such as adrenocorticotropic hormone (ACTH), which is released by the pituitary gland and is itself stimulated by corticotropin-releasing hormone (CRH), which is produced by the paraventricular nucleus (PVN) of the hypothalamus. The HPA axis is a key mediator of sickness metabolism following infection and drives an overall increase in systemic corticosteroid levels [117, 118]. These hormones were shown to help the immune response by promoting helper T-cell polarization and stimulating immune cell activity [119]. In addition, adrenal activity is crucial for maintaining a normal electrolyte balance and blood pressure during infection. People with adrenal insufficiency have a fourfold-fold greater chance of dying from infection, typically because of an adrenal crisis [120]. Immune-mediated regulation of the HPA axis is mediated both at the level of the hypothalamus [117] and the adrenal gland, and we will focus on the latter here.

Single-cell and spatial RNA sequencing studies have revealed that the adrenal gland is home to a specific pool of immune cells, dominated by T cells and macrophages, which are not equally distributed throughout this organ [121, 122]. Lymphocytes are preferentially found in the zona glomerulosa [123], CD115^+^MHC-II^Dim^ macrophages are observed at the corticomedullary junction, whereas classic MHC-II^bright^ macrophages are found throughout the adrenal cortex [124]. Endocrine cells from the adrenal gland, both in the cortex and medulla, express receptors for various cytokines, including IL-1β, IL-6 and TNF [125–127], and are therefore under direct control by the immune system. During homeostasis, the depletion of CD115 macrophages alters lipid metabolism in the adrenal gland, resulting in a decrease in aldosterone production, a hormone important for the regulation of blood pressure [124]. Following viral infection, for example, with CMV, immune cell-derived IL-6 was shown to stimulate glucocorticoid production in mice, reduce vasodilatation and prevent excessive inflammation [128–130]. Moreover, IL-6 deficiency impaired the adrenal response to *Klebsiella pneumoniae* infection, causing increased mortality due to systemic hyperinflammation [131]. In the context of HIV infection, IL-1β and IL-6 stimulation of the adrenal cortex was found to be associated with increased systemic cortisol levels [132]. Apart from preventing an adrenal crisis, these hormones were shown to mediate Th2 polarization of the CD4 + T-cell response [133].

The adrenal medulla appears to be mostly impacted by bacterial infection. During sepsis, stress hormones, such as norepinephrine and corticosteroids, promote hyperglycemia. These hormones, in parallel with the high levels of the proinflammatory cytokines IL-1β, TNFα, and IL-6, inhibit insulin sensitivity in metabolically active organs such as the liver [42, 134, 135]. Moreover, insulin production by β cells is reduced, which results in increased blood glucose levels. This stress-induced hyperglycemia is initiated to ensure that sufficient levels of glucose reach vital organs under conditions of reduced blood flow [42, 135, 136]. Stress-induced hyperglycemia is therefore a survival strategy of immediate benefit, despite its long-term detrimental impact on peripheral organs and its acute inhibitory effect on the function of innate and adaptive immune cells [91, 137]. In addition, norepinephrine counteracts the vasodilatation associated with septic shock, and its exogenous administration therefore greatly reduces mortality in this condition [138]. Thus, proinflammatory cytokines influence the function of endocrine cells in a dose- and tissue-dependent manner to increase survival.

## The liver

The liver is one of the master regulators of metabolic homeostasis in the body. The liver metabolizes virtually all nutrients from either the digestive tract or nutrient stores in the body into a form that can be utilized by other organs. As such, the liver maintains homeostatic values of carbon metabolites, including glucose, lipids, and cholesterol. In addition, the liver produces most plasma proteins, such as albumin, clotting factors, complement factors and apolipoproteins. Furthermore, the liver serves as a storage site for glucose, minerals (iron, copper) and vitamins (A, B12, D, E, K). Finally, the liver neutralizes toxins, metabolizes drugs and secretes bile in the digestive system [139, 140]. Most of these functions are executed by hepatocytes, which are the main parenchymal cells in the liver. These cells are organized in lobules, which are hexagonal structures in which mixed blood from the portal vein and hepatic artery flows through sinusoid capillaries from the periphery toward a central venule [141]. Liver sinusoid endothelial cells (LSECs) are separated from hepatocytes by the very small space of Disse, which contains stem cell-like hepatic stellate cells (HSCs) important for the maintenance of hepatic connective tissue structure and for the storage of vitamin A [142, 143].

Finally, the liver is home to a wide variety of immune cells. The liver is continuously exposed to microbial components leaking from the gut, and its immune system is therefore poised to eliminate these threats without inducing excessive inflammation [144]. Moreover, these immune cells are instrumental for mediating changes in the homeostatic function of the liver in the context of sickness metabolism [42, 140]. This process is controlled either directly through cytokine-mediated modulation of hepatocyte activity or indirectly through the adaptation of endocrine regulation of liver function. Changes in blood carbon species, most notably glucose levels, are some of the best-studied metabolic adaptations to infection mediated by the liver. In response to fasting, the liver releases glucose by degrading its glycogen stores through gluconeogenesis. The extent of glucose release is controlled by the reciprocal action of the pancreatic hormones insulin and glucagon [32]. Following strong infection, the synergistic action of IFNγ and IL-1β on pancreatic β cells leads to an increase in insulin production that exceeds the levels required to compensate for skeletal insulin resistance. As a result, hepatic glycogenolysis is impaired, leading to a reduction in fasting plasma glucose levels [47]. Systemic glucose restriction strengthens IFN-I responses to viral infection by curtailing cellular lactate production and consequently inhibiting MAVS and NF-kB signaling [45]. Under extremely high pathogenic loads, for example, during sepsis or severe SARS-CoV-2 infection, the adrenal glands produce stress hormones such as epinephrine, norepinephrine and cortisol. These molecules overrule the normal glycemic control mediated by insulin, inhibit glycogen synthesis and promote gluconeogenesis, resulting in stress-related hyperglycemia [145, 146]. Moreover, high concentrations of TNF, IL-1β and IL-6 in circulation cause insulin resistance in hepatocytes and skeletal muscle cells [147] and inhibit insulin production by the pancreas [148]. The purpose of stress-induced hyperglycemia is to ensure that systemic glucose levels are sufficient to preserve vital organ functions under extreme conditions [145].

Cytokines also directly impact hepatocyte metabolism, which has been best characterized for the acute phase response in the context of bacterial infection. In response to several different cytokines, but most notably to IL-6 and IL-1β derived from monocytes and macrophages in the liver, hepatocytes start producing ~30 acute phase proteins (APPs) which have various antimicrobial properties. These include C-reactive protein, haptoglobin, serum amyloid A, fibrinogen, a1-atitrypsin, antichymotrypsin, a1-acid glycoprotein, ceruloplasmin and complement factor B. At the same time, the production of other factors, such as albumin, transferrin, fibronectin, a-fetoprotein and complement factor 3, is reduced [149–153]. APPs strongly impact systemic inflammation and pathogen clearance. APPs act through a wide range of mechanisms, including the destruction and growth inhibition of pathogens, blood coagulation, pyrogenesis and the promotion of immune cell infiltration into the site of infection. [154–156]. One APP with a prominent metabolic effect is hepcidin, which regulates iron levels in serum. Iron is a vital micronutrient for both human and microbial cells and is carefully chaperoned by proteins such as transferrin, lactoferrin and ferritin under normal conditions. Upon infection, hepcidin is produced, which stimulates iron sequestration in macrophages, thus reducing iron availability to microbes [157]. Hepcidin-deficient mice therefore exhibit significantly increased mortality following infection with the siderophilic bacterium *Vibrio vulnicus* [158]. Low iron and increased hepcidin levels in serum were found to be associated with increased survival in critically ill human sepsis patients [159].

Proinflammatory cytokines also affect the metabolic functions of the liver associated with amino acids [160–162]. Following acute infection, the T helper 1 (Th1)-type cytokines IL-1β and TNF cause an increased influx of amino acids into the bloodstream by promoting protein degradation in skeletal muscle [163]. At the same time, these proinflammatory mediators promote amino acid uptake by the liver, thus maintaining systemic homeostasis [164–166]. In vitro experiments have shown that IL-6 stimulates hepatocytes to take up amino acids directly, whereas TNF indirectly mediates this effect by promoting glucagon secretion by pancreatic α-cells [167, 168]. This shift in amino acid metabolism promotes the synthesis of specific molecules involved in the antipathogenic response. By altering amino acid availability, IL-1β, TNF, IFNγ and TGFβ promote the synthesis of a narrow spectrum of APPs, such as complement factor B and complement component 3 [169–175], which are essential for protection against bacterial infection [176]. In addition, IFNγ produced by intrahepatic T cells promotes the expression of indoleamine 2,3-dioxygenase (IDO), which converts tryptophan to kynurenine, both in immune cells and hepatocytes [177, 178]. IDO-mediated tryptophan consumption reduces the replication of intracellular pathogens, including hepatitis B virus, *Toxoplasma gondii* and *Leishmania donovani* [179, 180]. At the same time, kynurenine and its downstream metabolites inhibit excessive activation of immune cells such as T cells, dendritic cells, monocytes and macrophages, thus reducing immunopathology following infection [181]. IDO deficiency therefore increases protection against lethal influenza infection [182].

In summary, the modulation of liver metabolism plays a key role in the execution of sickness metabolism, and its abrogation therefore negatively impacts the response against infection.

## The nervous system

**Fig. 2:**
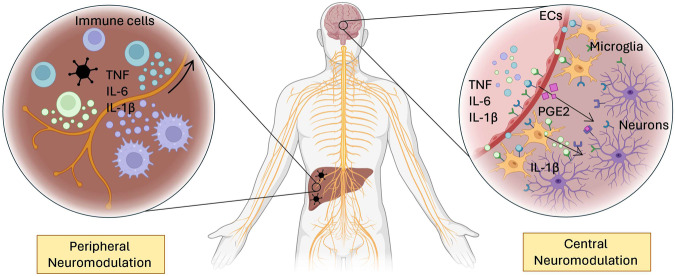
Neuromodulation by the immune system. Although the nervous system is immune-privileged, it is not devoid of immunological control. Cytokines impact the central control of metabolism via cellular intermediates. (Left) Peripheral neuromodulation is mediated by neurons of the *N. vagus*, which are under the direct influence of cytokines produced in the periphery. These signals are sent to the hypothalamus, resulting in central regulation of sickness metabolism. (right) Central neuromodulation occurs directly in the brain. Immunological mediators can cross the endothelial barrier in the hypothalamus and reach microglia. These cells produce cytokines locally, thus amplifying the signal toward neurons. Endothelial cells (ECs) in the hypothalamus also sense cytokines and, in response, produce molecules such as PGE2. This leukotriene is sensed by neurons and mediates metabolic changes such as alterations in body temperature. Made with biorender.com

The central nervous system (CNS), particularly the hypothalamus, plays a central role in metabolic homeostasis [183]. A detailed description of the neuronal circuits in the hypothalamus and how they modulate systemic metabolism is described elsewhere [184] and will not be provided here. Mounting evidence suggests that proinflammatory cytokines mediate sickness metabolism by acting on hypothalamic brain areas [185]. How this process is mediated has long remained a matter of debate considering the long-standing dogma that the brain is an immune-privileged site protected by the blood–brain barrier. Experimental evidence indicates that activated immune cells can impact the brain in two ways (Fig. 2). The first route is via a neural intermediate of the peripheral nervous system. Cytokines produced at the site of infection activate sensory neurons that inform higher brain areas through the *Nervus vagus* [186, 187]. The *N. vagus* innervates almost all tissues that serve as entry points for pathogens, such as the lungs, the mucosa of the gastrointestinal tract and the abdominal organs [188]. Moreover, the sensory branches of the *N. vagus* express receptors for proinflammatory cytokines [189]. It was shown that the injection of proinflammatory cytokines or LPS or infection with *Salmonella typhimurium* induces a loss of appetite, i.e., anorexia, by increasing the activity of the *N. vagus*. Moreover, vagotomy prevented the development of infection-induced anorexia upon LPS treatment [190–193]. Recently, it was shown that intraperitoneal injection of IL-1β mediates insulin secretion through activation of the *N. vagus*. Thus, immunomodulation of the peripheral nervous system by immune cells in infected tissues impacts the central regulation of systemic metabolism [194]. The purpose of this mechanism is to reduce the pathogenic burden. Indeed, when *N. vagus*-mediated sickness behavior was prevented by force-feeding anorexic mice, the pathogenic load strongly increased in mice infected with *Salmonella typhimurium* [193]. Interestingly, whereas the *N. vagus* innervates a large number of organs in the body, localized mild infection in one of these organs does not lead to sickness metabolism. One possibility is that proinflammatory cytokines need to reach defined threshold values to induce changes in centrally regulated metabolism.

A second method for immune cells to modulate hypothalamic activity is through cellular intermediates within the hypothalamic centers for energy homeostasis. Whereas the blood‒brain barrier is a tight protective layer in most parts of the brain, this is not the case in the hypothalamus. In this region, the blood‒brain barrier is modified to allow fast and dynamic passage of hormones and metabolites. This is required for their detection by neurons in the hypothalamus, which subsequently regulate their values in the blood by sending activating or inhibitory signals to the organs that produce them [195]. In addition, active systems that transport cytokines such as IL-1β, IL-6 and TNF into the brain have been identified [196]. Both bacterial and viral infections therefore have been shown to increase the levels of proinflammatory cytokines in the CNS although the brain itself is rarely invaded by these pathogens [197–200].

The hypothalamus can be subdivided into several anatomical subregions, including the PVN, the preoptic area (POA) and the arcuate nucleus, which contain pro-opiomelanocortin (POMC), neuropeptide Y (NPY) and agouti-related peptide (AgRP) producing neurons that reciprocally regulate energy homeostasis. It was shown that intracranial injection of TNF activates the NF-kB signaling pathway predominantly in POMC neurons and to a much lesser extent in NYP or AgRP neurons. This selective activation of the NF-kB signaling pathway in specific neurons leads to increased food intake and obesity [201]. In contrast, the deletion of IKKβ in POMC neurons prior to LPS injection prevented the development of anorexia [202]. Moreover, specific deletion of IKKβ in AgRP neurons only partially protected mice from obesity. The infusion of IL-1β, TNF or IL-2 stimulates the PVN, resulting in an increase in CRH in circulation, the activation of the HPA axis and an increase in serum glucocorticoid levels [203–206]. The infection of mice with Newcastle disease virus (NDV) resulted in an increase in plasma corticosterone levels, which was not observed in hypophysectomized animals. The infusion of mice with a supernatant of NDV-infected cell cultures also increased ACTH levels in the blood, which was prevented in the presence of IL-1β-neutralizing antibodies. Systemic injection of LPS was shown to inhibit GnRH neurons in the PEO in an IL-1β-dependent manner, leading to a repression of sex behavior[207]. Other aspects of centrally regulated sickness metabolism were shown to be impacted by infection in a similar manner [207]. Importantly, hypophysectomy greatly reduces the survival of rats exposed to endotoxic shock and of mice infected with poliomyelitis virus [208, 209], which could be prevented by exogenous administration of glucocorticoids.

The impact of the immune system on centrally mediated sickness metabolism does not operate exclusively to alter the HPA axis. For example, infection is well known to cause an increase in sleep [210]. Cytokines such as IL-1β and TNF produced following infection were shown to directly inhibit [211] the dorsal raphe nucleus in the brain, which is responsible for wakefulness and to activate the POA/basal forebrain region that promotes nonrapid eye movement sleep [212]. This type of sickness metabolism promoted the antipathogenic response, as sleep disruption in mice during infection with malaria significantly impaired survival [213]. In addition, sleep disruption in humans is associated with an increased incidence of infection [214]. Mice with neuron-specific deficiency of AcP, an essential component of IL-1 signaling in the brain, fail to experience increased sleep following influenza infection, indicating that sleep is immune-mediated and results in an increased mortality rate [210]. In summary, cytokines can mediate sickness metabolism by stimulating the central nervous system, and the characteristic of the response is dictated by the group of neurons that is activated [215, 216].

Cytokines derived from the periphery do not stimulate neurons directly; an amplification step via cellular intermediates in the hypothalamus is required. Myd88 is a key intracellular mediator of signaling following LPS stimulation of toll-like receptor 4. When wild-type bone marrow was transplanted into Myd88-deficient animals, the proinflammatory response to LPS injection was restored in the periphery but did not lead to cytokine production in the CNS. Consequently, these mice were protected from LPS-induced anorexia [217, 218]. Thus, cytokines from the periphery do not target neurons directly but rather use a glial intermediate. Indeed, cytokines such as IL-1β, IL-6 and TNF were shown to be produced in the brain itself following peripheral viral infection, most notably by microglia [219, 220]. Microglia are tissue-resident macrophages that play important defensive roles against pathogens and ameliorate tissue damage. Furthermore, this cell subset is important for maintaining tissue homeostasis in the brain. As such, they have been shown to play crucial roles in neurogenesis and in the remodeling of neuronal circuits [221, 222]. Upon infection, these cells are the major source of proinflammatory cytokines, and their depletion prevents CMV-mediated neural pathology in a model of congenital infection [223]. In addition to microglia, endothelial cells in capillaries in the brain also play a crucial role in mediating sickness metabolism, especially with regard to temperature regulation. Cytokines derived from the periphery bind to these endothelial cells and promote the synthesis of cyclo-oxygenase 2 (COX2). This enzyme is responsible for the conversion of arachidonic acid into prostaglandin E2 (PGE2). Endothelium-derived PGE2 targets adrenergic neurons of the sympathetic nervous system in the hypothalamus [224, 225]. Through a neurological circuit reaching into the periphery, these cells stimulate heat generation in brown fat and cause vasorestriction to prevent passive heat loss through the skin [84]. An increase in core temperature does not appear to have a major impact on pathogen replication [226] but rather increases the immune response against infection [23]. For example, CD8 T cells cultured at 39 °C showed increased effector potential due to increased mitochondrial metabolism [227] and promoted CD4 helper T-cell polarization [228]. Animals deficient for COX2 do not exhibit a febrile response to inflammatory stimuli [229]. Not surprisingly, animals deficient for COX2 showed a strong increase in viral titers in the lung after infection with influenza A virus, whereas the concentrations of cytokines such as TNF, IL-1β, IL-6 and IFNγ were reduced in bronchoalveolar lavage fluid [230]. Several pyrogenic cytokines, including IL-1β, TNF and IL-6, can promote PGE2 production by endothelial cells, and the latter appears to be of key importance [84]. IL-6 deficiency in mice makes these animals resistant to LPS-induced hyperthermia [231]. Moreover, intracranial injection of IL-6, but not of IL-1β, in these animals restored the febrile response in *Il6*^*−/−*^ mice [231].

In conclusion, immune cells and immune-derived mediators have a major impact on neuronal control of metabolic homeostasis. In the context of infection, inflammatory signals from the periphery are filtered through peripheral neurons, tissue-resident glial cells and endothelial cells, thus allowing carefully regulated exposure of neurons of the CNS to these signals.

## Sickness metabolism as a source of metabolic disease

**Fig. 3:**
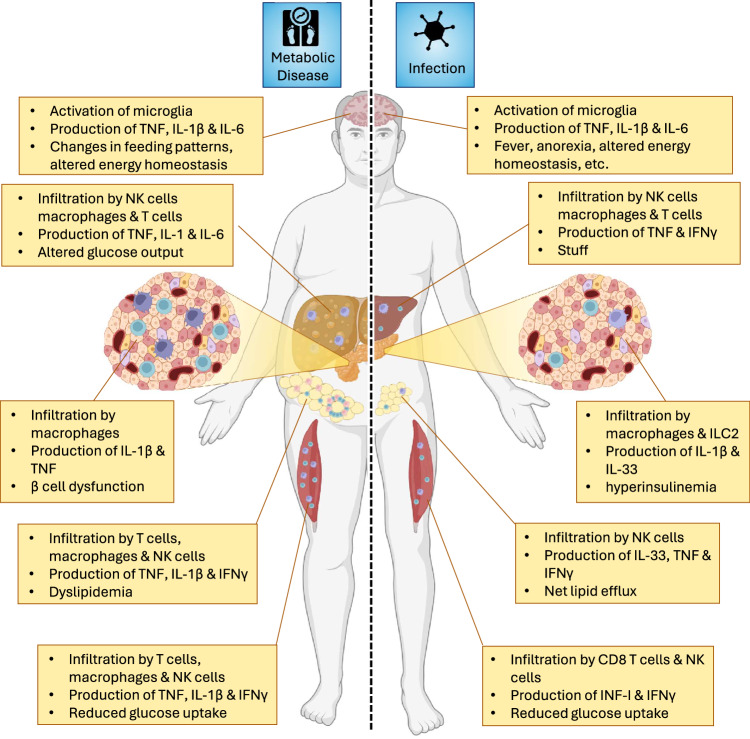
Parallels between sickness metabolism following infection and in the context of metabolic disease. Obesity has a broad, detrimental impact on systemic metabolism and greatly increases the risk of developing metabolic syndrome (MetS). The immune system plays a role in the pathophysiology of most organs affected by MetS. The immune-mediated changes observed in the metabolically stressed organs are similar to those of some of the alterations observed after infection. This finding supports the hypothesis that metabolic disease is partially caused by a derailed immunological response against tissues in which metabolic stress is perceived as an infection. This dysregulated immune response leads to chronic metabolic changes normally associated with sickness metabolism. Made with biorender.com

Metabolic diseases are pathological conditions in which systemic metabolism is negatively impacted. With an increasingly overweight global population, obesity is by far the most common cause of metabolic aberrations and causes symptoms such as visceral obesity, hypertension, hyperglycemia, and dyslipidemia. If three or more of these symptoms occur in the same patient, we speak of metabolic syndrome (MetS) [232, 233]. MetS has a major negative impact on the wellbeing of people and is therefore a great concern for public health [234]. MetS primarily has metabolic causes but also has a clear immunological component [140, 235]. Interestingly, immunological changes in the organs of people with MetS are strikingly similar to those observed in individuals after infection (Fig. 3), which we will briefly describe in the following subsections.

### Adipose tissue

WAT is well known to play a key role in MetS, especially for the development of type 2 diabetes (T2D), a disease defined by an inability to maintain blood glucose levels below well-specified threshold values [236]. The accumulation of fat in adipose tissue causes sterile inflammation marked by the influx of immune cells [26]. Patients with T2D therefore have increased levels of proinflammatory cytokines in circulation, resembling a chronic low-grade infection [7, 93, 237] of a Th1 type, which is typically associated with viral infection. In response to metabolic stress, hypertrophic adipocytes induce the surface expression of ligands for the stress receptor Ncr1. This signal is perceived by natural killer cells, which drive the polarization of tissue-resident macrophages from an M2- to an M1-like phenotype [238]. These cells in turn promote the influx of other proinflammatory cells, including Th1 CD4 + T cells, CD8 + T cells and B cells. Cytokines produced by these cells, such as TNF, IFNγ and IL-1β, reach the bloodstream and contribute to the development of systemic insulin resistance (IR) [7, 26]. Strikingly, this systemic IR is also observed upon viral infection, both in humans and mice [6, 239]. Thus, metabolic stress in obese adipose tissue causes an immune response and associated sickness metabolism that is normally induced by viral infection. As the cause of inflammation is never resolved in obesity, the metabolic changes that are transient in viral infection become permanent.

### Skeletal muscle

Mounting evidence suggests that inflammation within skeletal muscle plays a crucial role in the pathology of metabolic syndrome [240]. This role has been most extensively studied in dietary models in which animals are fed food in which a large fraction of calories are derived from animal fat. Using this diet-induced obesity (DIO) model, various immune cell subsets, most notably macrophages and T cells, infiltrate skeletal muscle [241–243]. These immune cells are primarily located inside visceral adipose tissue that surrounds and penetrates myofibrils [241]. In situ, immune cells produce proinflammatory cytokines, such as TNF, IL-1β and IFNγ [243–245]. Skeletal muscle inflammation impairs insulin signaling and promotes the development of insulin resistance [241, 246]. Since skeletal muscle is a major sponge for glucose following a meal, DIO-induced inflammation leads to hyperglycemia and promotes the development of type 2 diabetes (T2D) [247]. Notably, IR in skeletal muscle is mediated by NK cells, and these immunological mediators also contribute to muscle IR in the context of viral infection [6].

### Pancreas

The role of the tissue-resident immune system in mediating type 1 diabetes (T1D), a metabolic autoimmune disease in which pancreatic β cells are specifically eliminated, has long been established [248]. In T1D, islet-resident antigen-presenting cells (APCs), such as macrophages and dendritic cells (DCs), activate the adaptive immune system, which mediates the destruction of β cells. The depletion of islet macrophages prevents the development of T1D, as the priming of autoreactive CD8 + T and B cells within pancreas-draining lymph nodes is impaired [249]. This impairment was shown to be dependent on antigen presentation, as the excision of draining lymph nodes prevented the development of insulitis as well as T1D [250]. Importantly, several viral infections were shown to trigger the development of T1D [251], supporting an overlap between tissue-resident immune responses in the context of viral infection and metabolic disease. Studies in the Langerhans islets of patients with T2D have shown that during the development of obesity, there is a significant increase in proinflammatory TNF-α- and IL-1β-producing M1-like macrophages. The depletion of these macrophages was associated with increased insulin production by pancreatic β cells [252].

### Brain

Microglia have been shown to be important in the pathophysiology of metabolic disorders. Microglia respond not only to proinflammatory cytokines but also to changes in the concentrations of various metabolites in the blood. These metabolites include saturated fatty acids, which are typically present at higher levels in the serum of obese individuals and animals in models of obesity [253–255] but also increase following infection [256, 257]. Metabolite-induced activation of microglia activates the NF-κB signaling pathway and promotes the polarization of these cells toward an M1-like phenotype. The subsequent release of proinflammatory cytokines such as TNF, IL-1β and IL-6 by these cells inside the mediobasal hypothalamus (MBH) modulates neuronal circuits important for metabolic homeostasis. Evidence that supports this theory comes from experiments in which researchers depleted microglia in the hypothalamus, which decreased hypothalamic inflammation and ameliorated obesity-related symptoms [258, 259]. Similarly, intracranial administration of IL-4, a cytokine that promotes polarization toward an anti-inflammatory M2-like phenotype, was found to inhibit weight gain [260]. This finding demonstrated that M1-polarized microglia have a pleotropic effect on the control of metabolic homeostasis. However, exactly how obesity-mediated activation of microglia modulates neuronal circuits in the hypothalamus remains to be investigated.

### Liver

The liver is the main site of endogenous glucose production [240]. As in adipose tissue, obesity leads to the accumulation of proinflammatory M1-like macrophages in the liver [261] which activate proinflammatory signaling pathways in hepatocytes, thereby causing hepatic insulin resistance [262, 263]. Inadequate insulin-induced inhibition of hepatic glucose production leads to an increase in fasting plasma glucose concentrations and contributes to the development of T2D [240]. In addition, metabolic stress in hepatocytes activates innate immune cells in the liver, leading to the development of metabolic dysfunction-associated liver disease (MAFLD) [264]. Upon excessive caloric intake, hepatocytes accumulate fat droplets. This fat accumulation leads to the upregulation of so-called stress ligands for the activating immune receptor NKG2D [265] on γδ T cells and CD4^-^CD8^-^ double-negative T cells [266, 267]. A similar stress response is induced by infection, for example, by cytomegalovirus infection [268]. Upon their activation, innate immune cells mediate the recruitment of myeloid cells. These include proinflammatory M1-like macrophages, which produce TNF, IL-1β and IL-6, in the context of both metabolic disease and viral infection [265, 269]. In addition, these myeloid cells recruit cells of the adaptive immune system. The subsequent inflammatory milieu in the liver caused by metabolic stress contributes to hepatocyte death, hepatic stellate cell activation and fibrosis [264].

Thus, the tissue-resident immune system plays a key role in metabolic disease, and the systems it deploys appear to be derailed antipathogenic defense mechanisms.

## Concluding remarks

Homeostasis plays a crucial role in maintaining optimal organ function by ensuring that metabolites remain within well-defined threshold levels under most conditions. However, during infection, homeostatic values can be temporarily altered, meaning that various tissues do not operate under ideal conditions which makes us feel sick. However, the acute, life-threatening danger posed by pathogens justifies that organs temporarily function suboptimally. Nonetheless, this strategy has risks, and the degree of metabolic modulation must be carefully balanced against the threat posed by the pathogen. Clinically noticeable changes in systemic metabolism therefore occur only when the infection reaches a significant degree of severity. Nevertheless, many questions remain unanswered with regard to sickness metabolism. The adaptations our metabolism undergoes during infection appear to be finely tuned to the specific nature of the invading pathogen, yet the mechanisms responsible for this specificity remain unclear. This puzzle is particularly intriguing given that cytokines—such as TNF, IL-6, IFNγ, and IL-1β—seem to play a role in most of the metabolic changes associated with sickness metabolism. Therefore, an additional layer of regulation must exist, likely involving tissue-specific factors and immune cells. Identifying these factors is crucial because they appear to play a major role in the development of metabolic diseases. By unraveling the mysteries of why we feel sick, we can pave the way toward better health.
